# Male Breast Cancer: Reevaluate Our Opinion

**DOI:** 10.1155/2020/6245415

**Published:** 2020-02-06

**Authors:** Santosh Kale, Rajmohan Rammohan, Vilma Vas, Chris Elsayad

**Affiliations:** Department of Internal Medicine, Nassau University Medical Center, East Meadow, Nassau County, NY, USA

## Abstract

Male breast cancers (MBCs) are relatively uncommon malignancy with less than 1% incidence. MBC presents at a later age with a more advanced presentation as compared to the female breast cancer. Due to the paucity of the number of cases and trials regarding the MBC, female breast cancer treatment protocols are applied. Mastectomy and hormonal therapy remains the mainstay of treatment. Moreover, the data about prognosis of MBC remains limited.

## 1. Introduction

Breast cancer is the most common malignancy in women [1]. Breast cancer in females is diagnosed around the 6th to 7th decade of life, but the diagnosis of breast cancer in a middle-aged adult male is very rare [2]. The age of incidence of breast cancer in the males tend to be higher than the females and is attributed to delayed reporting in males. The following is a case presentation of MBC after surgery and chemotherapy followed by radiation therapy. The patient is currently on Tamoxifen and presented to the primary care clinic for routine follow-up.

## 2. Case Presentation

**Figure 1:**
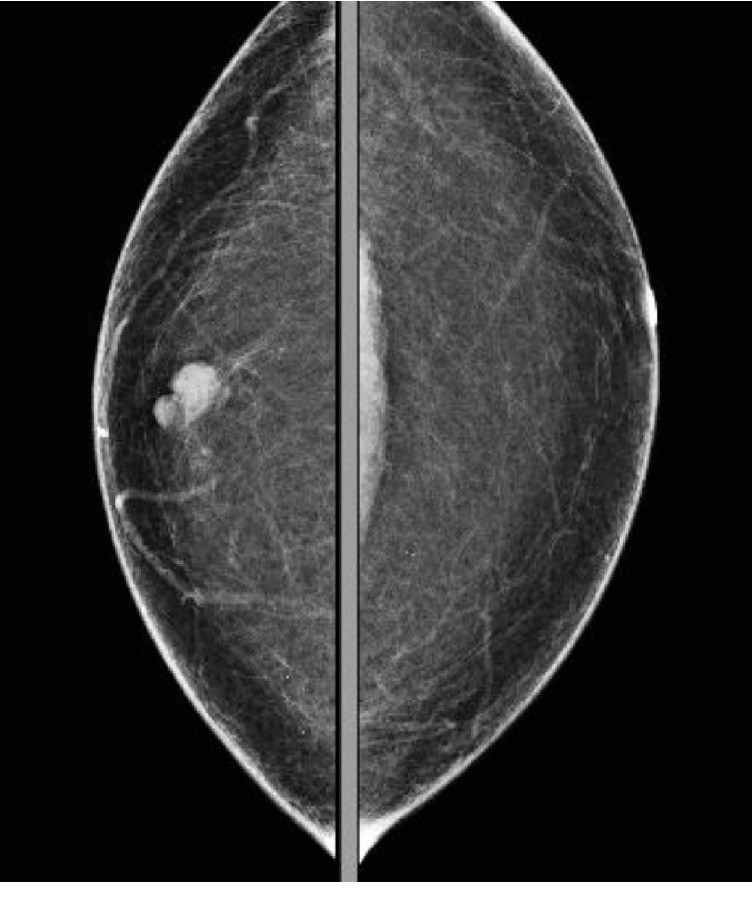
A 1.2 cm lobulated mass in the right retro areolar breast.

**Figure 2:**
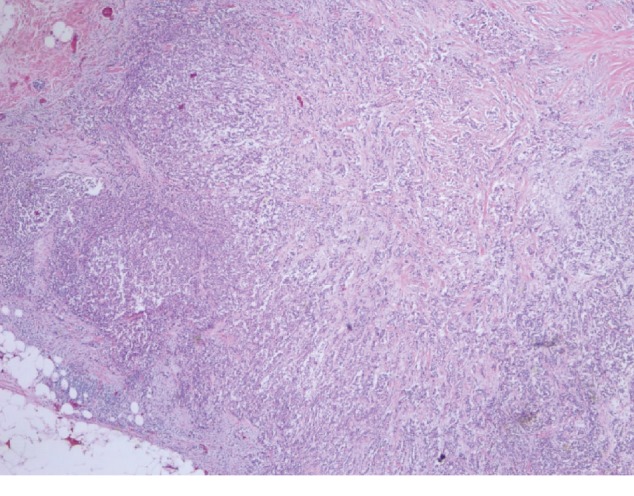
Right breast biopsy positive for invasive ductal carcinoma at 2.5x power field.

**Figure 3:**
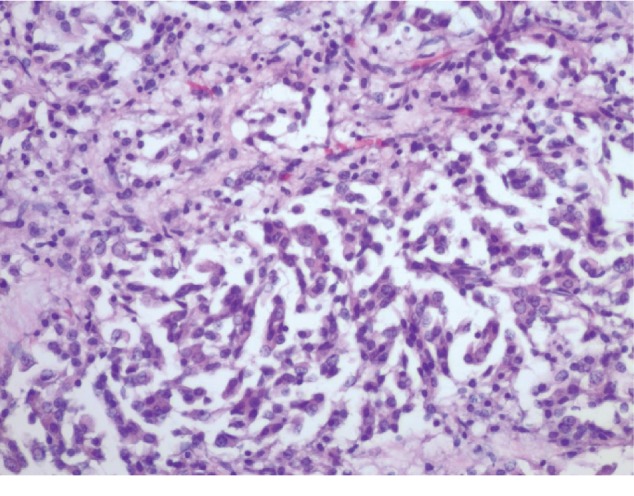
Right breast biopsy positive for invasive ductal carcinoma at 20x power field.

**Figure 4:**
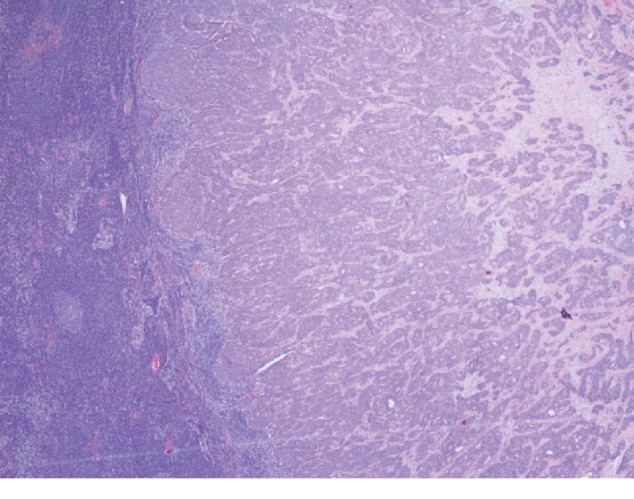
Biopsy of the right lymph node with metastatic carcinoma.

A 44-year-old male presented in our primary care clinic for routine follow-up. The patient had been noticing a lump in the right nipple for 4 years associated with 10/10 intermittent sharp pain worse at night and relieved with cold pack. The patient's mother died from breast cancer at the age of 47. She was diagnosed at age 40 and survived for 7 years post diagnosis. The patient has 3 sisters, and no other family history of breast cancer. His mammogram showed a 1.2-centimeter (cm) lobulated mass in the right retro areolar breast (Figure 1). The core needle biopsy was positive for invasive ductal carcinoma estrogen receptor (ER+) and progesterone receptor (PR+) and negative for human epidermal growth factor receptor 2 (HER–) (Figures 2 and 3). MBC often belong to the subtype present in our patient [3]. The patient underwent simple mastectomy and right sentinel lymph node biopsy. The patient was also given dose-dense adjuvant doxorubicin and cyclophosphamide (DDAC) and Taxane chemotherapy which was stopped after 3 doses of the Taxanes as it was complicated with pneumonitis. The patient's lymph node biopsy came back positive for metastatic carcinoma, and the patient received radiation therapy (Figure 4). The tumor was classified as stage IIA. Eventually, the patient was started on >5 years course of Tamoxifen therapy which is currently being tolerated well. The annual mammographic screening and clinical exam of the contralateral breast were normal.

**Figure 5:**
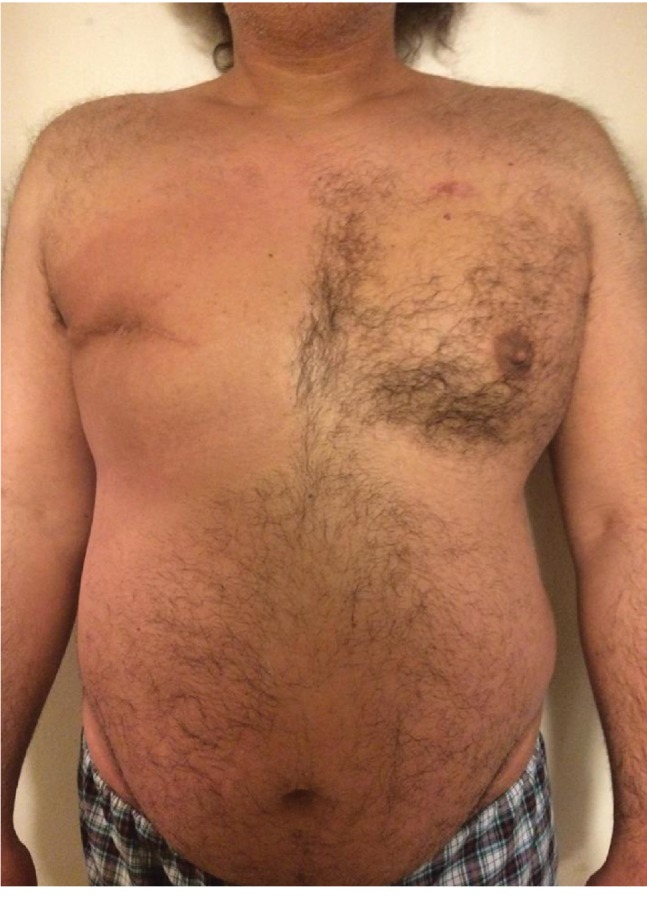
Front view of the old healed long lateral scar following mastectomy on the right chest.

**Figure 6:**
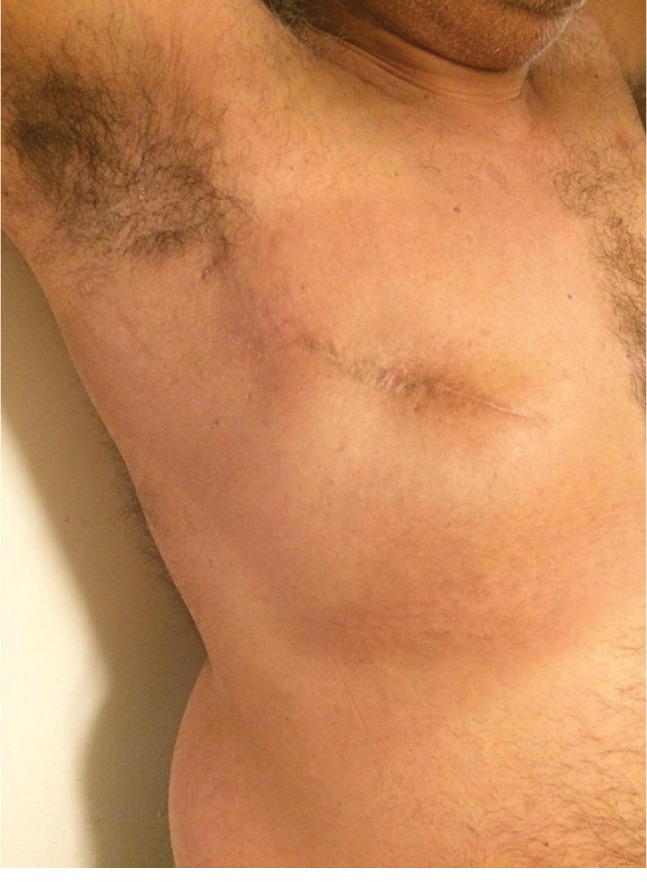
Right diagonal view of the old healed long lateral scar following mastectomy of the right chest.

He denies any active complaints 3 years post diagnosis except for some concerns of trouble maintaining erections and weight gain likely secondary to the effect of Tamoxifen. Physical examination was positive for old healed long lateral scar on the right chest (Figures 5 and 6). Furthermore, he underwent genetic testing which revealed no evidence of BRCA mutations or other evidence of familial breast cancer.

## 3. Discussion and Conclusion

The aim of this article is to bring to the notice of the readers that breast cancer in males is a rare but possible diagnosis. Proper management can provide a good outcome and the patients can live a healthy life. Diagnostic workup and management protocols overlap with that of the female breast cancer management due to the lack of prospective clinical trials for the MBC [4]. Limited information and awareness about MBC can lead to unexpected outcomes. Age, genetic factors, and obesity are certain risk factors for MBC [5]. BRCA 1 and BRCA 2 genes are the most common high-risk genes implicated in breast cancer, and thus screening is recommended in particular cases with family history of breast or ovarian cancer [6]. Nevertheless, as per literature, the prognosis of the breast cancer in females and males remains the same [7]. Lymph node-positive breast cancer offers a worse prognosis. We present a case of MBC with sentinel lymph node positive for metastatic carcinoma. Our patient did not have any recurrences post treatment and was able to return to routine life with no limitations. We attempt to educate our readers about MBC and its management. As a common perception, MBC does not always lead to poor prognosis. Continuous advancement in diagnosis and management of MBC should lead to better prognosis in coming years.

Tamoxifen is an ER receptor antagonist which is commonly used after primary treatment for ER+ breast cancer as it has shown to improve the survival [8, 9]. Adverse effects of tamoxifen therapy included loss of libido, weight gain, and hot flashes, and our patient had symptoms in consistent with these. Studies evaluating the adverse effect of the tamoxifen therapy in MBC are limited. This article aims to highlight these effects as well.
